# Cross-Sectional Analysis of Psychological Mediators Between Occupational Trauma and PTSD in Metropolitan Firefighters

**DOI:** 10.3390/ejihpe15050075

**Published:** 2025-05-09

**Authors:** Ahmet Erhan Bakirci, Vedat Sar, Ali Cetin

**Affiliations:** 1 Department of Civil Defense and Firefighting, Vocational School of Technical Science, Istanbul University-Cerrahpasa, 34320 Istanbul, Turkey; 2 Department of Psychiatry, Faculty of Medicine, Koc University, 34450 Istanbul, Turkey; vsar@ku.edu.tr; 3 Department of Obstetrics and Gynecology, Faculty of Medicine, University of Health Sciences Hamidiye, 34668 Istanbul, Turkey; ali.cetin@sbu.edu.tr

**Keywords:** post-traumatic stress disorder, screening, childhood trauma, depression, dissociation, suicidality, occupational trauma, firefighters

## Abstract

Objective: The present investigation sought to examine the interrelationships between early-life adverse experiences, dissociative symptoms, suicidal ideation, and depressive manifestations among metropolitan firefighters screened with post-traumatic stress disorder (PTSD), and to elucidate the potential mediating effects of these psychological variables on both the presence and severity of PTSD symptomatology in this high-risk occupational cohort. Methods: A cross-sectional investigation was performed to assess psychological conditions among 760 metropolitan male firefighters, employing conditional process analysis with multiple mediation modeling (PROCESS macro Model 6). The investigative protocol employed validated psychometric instruments including the PTSD Checklist (PCL-5); the Childhood Trauma Questionnaire (CTQ-33); the Dissociative Experiences Scale (DES); the Suicidal Behaviors Questionnaire (SBQ-4); and the Patient Health Questionnaire (PHQ-9). Bootstrap resampling (*n* = 5000) generated bias-corrected 95% confidence intervals, enabling interrogation of complex trauma response mechanisms. Results: Conditional process analysis demonstrated that childhood trauma functions as a significant mediator (indirect effect = 0.142, 95% CI [0.086, 0.198]), with emotional abuse pathways revealing significant mediational effects (β = 0.285, *p* < 0.001). Stratifying participants using a PCL-5 ≥ 33 threshold (non-PTSD: *n* = 543, 71.5%, median PCL-5: 22; PTSD: *n* = 217, 28.5%, median PCL-5: 39), the investigation elucidated serial mediation mechanisms, particularly through childhood trauma to dissociative experiences (serial indirect effect = 0.168, 95% CI [0.092, 0.244]), collectively explaining nearly half of PTSD variance through complex psychological interaction pathways. Conclusions: Conditional process analysis revealed childhood trauma as a pivotal mediator, with emotional abuse pathways demonstrating significant mediational effects, while dissociative experiences emerged as a significant secondary mechanism, collectively explaining a substantial portion of PTSD variance through interactions between occupational trauma exposure and intrinsic psychological vulnerabilities.

## 1. Introduction

The systematic investigation of trauma response patterns among emergency service personnel reveals increasingly complex trajectories between occupational exposure and psychological burden. Metropolitan firefighters, operating at the nexus of acute crisis intervention and sustained emergency response, encounter several types of stressors that transcend conventional trauma paradigms. While their operational framework encompasses structured crisis protocols and team-based response mechanisms (Dautovich et al., 2022; Jones, 2017; Karnick et al., 2024; Lentz et al., 2022; Wolffe et al., 2023), the cumulative impact of recurrent trauma exposure manifests through intricate psychological cascades that extend beyond immediate stress responses. These cascades, characterized by complex interactions between occupational trauma and various psychological vulnerability dimensions, often crystallize into distinct clinical presentations, predominantly post-traumatic stress disorder (PTSD). Recent empirical evidence suggests that this relationship between occupational trauma and PTSD operates through sophisticated mediational pathways, incorporating both pre-existing vulnerability factors and emergent psychological states (Carleton et al., 2018; Cherry et al., 2021; Cramm et al., 2021; J. E. Kim et al., 2018; J. I. Kim et al., 2020; J. H. Lee et al., 2018; Oliveira et al., 2023; Teoh et al., 2019). This evolving understanding necessitates a more nuanced examination of the psychological mechanisms that modulate the translation of occupational trauma exposure into PTSD manifestation, particularly within the high-stakes operational context of metropolitan emergency services (Alshahrani et al., 2022; Cankardas & Sofuoglu, 2022).

The recognition of psychological vulnerability cascades in emergency service contexts, which necessitates a paradigmatic shift in our understanding of occupational trauma responses. Contemporary research increasingly acknowledges that the pathway from occupational trauma exposure to PTSD manifestation operates through multiple interwoven psychological mechanisms, each potentially serving as both mediator and moderator in the trauma response trajectory (Bevan et al., 2022; Cramm et al., 2021; Gulliver et al., 2021; Healy & Vujanovic, 2021; Henson et al., 2022; Karnick et al., 2024; Stanley et al., 2019). Metropolitan fire departments present a unique context where the cumulative burden of occupational stressors converges with pre-existing psychological vulnerabilities, compounding the risk of PTSD. The reluctance of emergency service personnel to engage with traditional psychological support systems, often stemming from concerns about operational fitness and professional stigma, adds another layer of complexity to this dynamic (Cherry et al., 2021; J. H. Lee et al., 2018; Teoh et al., 2019). Moreover, the traditional conceptualization of simple trauma responses fails to capture the complex and dynamic psychological architecture underlying PTSD development, where mediational and pathways dynamically interact to shape clinical outcomes (Boyd et al., 2018; Carleton et al., 2018; Cherry et al., 2021; Dautovich et al., 2022; J. E. Kim et al., 2018).

The empirical investigation of psychological vulnerability cascades in emergency service personnel, which necessitates particular attention to the multidimensional nature of trauma response architectures. Contemporary investigations increasingly recognize distinct psychological dimensions, including childhood trauma patterns, dissociative phenomena, affective dysregulation, and suicidal ideation, as critical modulatory elements in the occupational trauma–PTSD pathway (Boyd et al., 2018; Dye, 2018; Jones, 2017; Noor et al., 2019; Roth et al., 2022; Serrano-Ibáñez et al., 2023). These psychological dimensions, rather than functioning as isolated clinical entities, appear to operate through sophisticated interactive mechanisms that modulate trauma response trajectories. Specifically, the manifestation of PTSD symptomatology in firefighters demonstrates remarkable heterogeneity in its psychological architecture, with various vulnerability factors potentially serving as mediators in the trauma response cascade (Carleton et al., 2018; Cherry et al., 2021; Dautovich et al., 2022; Henson et al., 2022; Javidi & Yadollahie, 2012). The interplay between these psychological dimensions becomes particularly salient in metropolitan emergency service contexts, where operational stressors intersect with pre-existing vulnerability patterns through complex mechanistic pathways (J. I. Kim et al., 2018; Lushchak et al., 2023; Mureșanu et al., 2022).

The selection of childhood trauma, dissociative experiences, depression, and suicidal ideation as key factors in this investigation is based on substantial theoretical and empirical evidence. Childhood trauma has been established as a significant vulnerability factor that can sensitize individuals to later stress responses, potentially through alterations in stress-regulation systems (Dye, 2018; McLaughlin & Lambert, 2017). Dissociative phenomena are increasingly recognized as both a response to overwhelming trauma and a potential mechanism through which trauma affects psychological functioning (Boyd et al., 2018). Depression represents a common comorbid condition with PTSD that may both result from and exacerbate trauma responses (Gulliver et al., 2021). Finally, suicidal ideation represents a critical clinical outcome that has been linked to both trauma exposure and PTSD in first responders (Healy & Vujanovic, 2021; Noor et al., 2019).

Despite significant progress in understanding the trauma response mechanisms, substantial gaps remain in delineating the intricate pathways connecting occupational trauma to PTSD. Existing research often fails to integrate the simultaneous effects of mediators, such as childhood trauma, dissociation, depression, and suicidal ideation, within a unified analytical framework. These factors are frequently examined in isolation, neglecting their interactive effects and the potential for bidirectional influences. Furthermore, traditional approaches predominantly rely on cross-sectional designs that cannot capture the dynamic and evolving nature of trauma response trajectories over time. This lack of methodological integration limits the capacity of current models to fully represent the psychological complexity inherent in trauma-exposed populations, particularly metropolitan firefighters who face unparalleled operational demands (Cramm et al., 2021; Noor et al., 2019; Roth et al., 2022). Addressing these limitations, this study introduces a conceptual framework that combines conditional process modeling with statistical techniques to examine how these psychological factors interact as mediators. By doing so, it not only enhances theoretical coherence but also provides a more sophisticated understanding of PTSD pathways, with direct implications for tailoring targeted interventions and prevention strategies for high-risk occupational groups (Boyd et al., 2018; Cherry et al., 2021).

Based on the existing literature and theoretical frameworks, we propose the following hypotheses: (1) childhood trauma will significantly mediate the relationship between occupational trauma exposure and PTSD symptoms; (2) dissociative experiences will serve as a significant mediator in the trauma-PTSD pathway; (3) depression and suicidal ideation will demonstrate significant mediational effects in the occupational trauma-PTSD relationship; and (4) there will be a significant serial mediation pathway from occupational trauma through childhood trauma to dissociative experiences, which will collectively explain a substantial portion of PTSD variance.

This study seeks to fill these significant gaps by presenting a theoretically sound and methodologically suitable approach that incorporates multiple mediational analysis. By employing statistical techniques such as serial mediation and conditional process analysis, this research aims to elucidate the interactions among psychological factors—including childhood trauma, dissociative experiences, depression, and suicidal ideation—that shape the occupational trauma-PTSD trajectory. Sociodemographic data were collected to provide a comprehensive understanding of the participant group and to illustrate the prolonged exposure of firefighters to psychological trauma over time. However, these variables were not included in the core analyses to prioritize the clarity and interpretability of findings regarding the intricate interactions between psychological conditions and trauma response mechanisms. Unlike traditional simple models, this framework accounts for the multidimensional and dynamic nature of trauma responses, providing a more thorough understanding of the psychological dynamics at play. By focusing on metropolitan firefighters, this study bridges theoretical gaps in trauma response models and offers actionable insights for tailored intervention strategies. Specifically, it highlights the importance of addressing pre-existing vulnerabilities and emergent psychopathological states to mitigate the risk of PTSD. This framework not only advances theoretical understanding but also forms a basis for future research toward developing intervention strategies designed for high-risk occupations.

## 2. Materials and Methods

### 2.1. Study Design and Analytical Framework

This study used a cross-sectional design, incorporating conditional process analysis to examine the complex interrelationships between occupational trauma, PTSD, and potentially mediating psychological factors. The analytical framework was structured to capture both direct effects and indirect pathways through which occupational trauma might influence PTSD manifestation, with particular attention to the modulatory roles of childhood trauma, depression, dissociation, and suicidal ideation.

The study protocol implemented a comprehensive data collection strategy, beginning with an occupational trauma assessment via the Life Events Checklist for *Diagnostic and Statistical Manual of Mental Disorders, Fifth Edition* (LEC-5, *DSM-5*), followed by detailed PTSD-based evaluation using the PTSD Checklist for DSM-5 (PCL-5), and systematic assessment of potential mediating variables through validated psychometric instruments detailed below.

### 2.2. Participants

This research was executed in accordance with ethical standards subsequent to formal authorization granted by the Social and Humanities Research Ethics Committee of the Istanbul University-Cerrahpasa (Approval No: 2023/22, dated 3 January 2023), following authorization from the Istanbul Metropolitan Fire Department. The study population comprised firefighters operating in a metropolitan environment characterized by high urbanization density and extensive industrial development, presenting unique operational stressors.

From an initial pool of 830 firefighters who provided informed consent, 760 participants contributed to the complete dataset used in the final analysis. Seventy participants were excluded due to incomplete questionnaire responses, maintaining analytical rigor for the mediational analyses. The final cohort (*n* = 760) consisted exclusively of full-time firefighters with no secondary occupations. Research forms were distributed through the department’s institutional communication network using a multi-modal approach: institutional emails, strategically placed QR codes in common areas, and paper formats upon request. To ensure no duplicate responses, each participant received a unique identifier code that could only be used once across all data collection methods. This implementation strategy achieved a participation rate of 91.6% over a 45-day data collection period.

The participants were made aware of their ability to withdraw from the study at any time and without providing a reason, as required by the rules for ethical research. Among the data security precautions that were implemented was the use of encrypted digital storage, with access being limited to approved members of the study team. The study design and implementation adhered to “the principles outlined in the Declaration of Helsinki”, with particular emphasis on participant confidentiality and voluntary participation. After the purpose and procedures of the study were fully disclosed, informed consent was obtained from all participants.

### 2.3. Study Protocol

The following sociodemographic variables were collected: age (in years), marital status (single, married, divorced, widowed), education level (high school, undergraduate, graduate), and years of service as a firefighter.

#### 2.3.1. LEC-5 and PCL-5

The LEC-5 was used to assess exposure to potentially traumatic events throughout the respondent’s lifetime. The LEC-5 evaluates exposure to 16 events recognized as potential triggers for PTSD or distress, along with an additional item that addresses any other exceptionally stressful event not included in the initial 16 items (Weathers et al., 2013). All participants completed both the LEC-5 and the PCL-5, regardless of their reported trauma exposure.

The PCL-5 is a 20-item checklist developed to determine PTSD symptoms based closely on the Diagnostic and Statistical Manual of Mental Disorders, Fifth Edition (DSM-5) criteria (Weathers et al., 2013). The PCL-5 is employed as a self-report measure to evaluate PTSD symptoms that have occurred within the past month. Participants evaluate each item to reflect the extent to which they have experienced distress from DSM-5 PTSD symptoms associated with their most recent traumatic event.

The PCL-5 can be evaluated using various scoring methods. The total symptom severity score, which ranges from 0 to 80, is calculated by summing the scores of the 20 items. Preliminary findings indicate that a PCL-5 cutoff score of 33 may serve as a marker for probable PTSD within the study population, with elevated scores correlating with increased PTSD symptomatology. Boysan et al. (2017) conducted an adaptation study of the scale in our country, yielding reliable results for PTSD assessment.

#### 2.3.2. Childhood Trauma Questionnaire 33 (CTQ-33)

The CTQ-33 is a self-report instrument utilizing a Likert scale to assess five dimensions of childhood maltreatment: emotional abuse, physical abuse, sexual abuse, emotional neglect, and physical neglect, as well as overprotection and overcontrol (Şar et al., 2021). Items are evaluated using a 5-point frequency scale: 1 indicates never true, 2 indicates rarely true, 3 indicates sometimes true, 4 indicates often true, and 5 indicates very often true. The score for each subscale varies from 5, indicating no history of abuse or neglect, to 25, reflecting a very extreme history of abuse and neglect. The total score, obtained by summing all five subscales, ranges from 25 to 125, signifying the severity of overall maltreatment experienced by an individual in childhood. A 3-item minimization/denial (M/D) score, which ranges from 0 (indicating no minimization) to 3 (indicating substantial minimization), can be computed to identify a response bias that downplays the extent of childhood trauma experienced by the individual.

#### 2.3.3. Dissociative Experiences Scale (DES)

The DES was employed to assess dissociative symptoms. This instrument consists of 28 self-report items designed to evaluate the severity of dissociative experiences (Bernstein & Putnam, 1986). Each item can receive a score ranging from 0 to 100. The scale assesses the frequency of dissociative experiences in daily life, with participants asked to indicate how often they experience each dissociative phenomenon. The Turkish version demonstrates reliability and validity (Yargic et al., 1993). The Turkish version of the scale demonstrated strong reliability and validity, with scores exceeding the 30 cut-off point suggesting potential dissociation (Boysan, 2014).

#### 2.3.4. Suicidal Behaviors Questionnaire-4 (SBQ-4)

The validity and reliability study of the SBQ was conducted by Linehan and Nielsen (Linehan & Nielsen, 1981). Bayam et al. adapted the SBQ to a four-item version in Turkish (Bayam et al., 1995). Each of the four items in the SBQ-4 evaluates distinct aspects of suicidality. SBQ-4 comprises four items: The initial item, “Suicide ideation and/or suicide attempt”, pertains to lifelong suicidal behavior and contains six options. The Likert scale is used to evaluate it, with a range of 0 to 5. The second item, “Suicidal ideation over the past 12 months”, is composed of five items and pertains to suicidal impulses that have taken place within the past year. Scored as Likert type 0–4 points. Third item: “Threat of suicidal attempt” (two options). Responses that indicate “no” are granted a score of 0; responses that indicate “yes” are granted a score of 1. Fourth item: “Self-reported likelihood of suicidal behavior in the future”: This item is associated with suicidal ideation and intent in the future. Likert type 0–4 points are assigned to the five options. The lowest possible score from the suicidal behavior questionnaire is 0 and the highest possible score is 14. The total score is determined by the arithmetic sum of all scores. As the scores increase, the severity of the suicidal ideation increases.

#### 2.3.5. Patient Health Questionnaire 9 (PHQ-9)

A self-administered version of a diagnostic instrument, the PHQ-9, is employed to screen for depression and to evaluate the severity of depression (Kroenke et al., 2001; Sari et al., 2016). It has been extensively used and is considered one of the most well-validated depression scales in both clinical practice and adult research.

### 2.4. Statistical Analysis

Statistical studies were conducted with the modules of SPSS version 28 (USA) alongside PROCESS macro v4.2 for mediation analyses. The distribution of variables was assessed using Shapiro–Wilk tests, and homoscedasticity was tested using Levene’s test. Given the non-normal distribution patterns in the dataset, descriptive statistics were presented as median plus interquartile range (IQR) for numeric variables and as count plus percentages for categorical variables.

Comparisons of study data in the participants with or without PTSD were performed using Mann–Whitney U and chi-square tests for numeric and categorical variables, respectively. Effect sizes were computed as r coefficients for numeric variables and Cramer’s V for categorical comparisons. Statistical significance was determined at *p* < 0.05, with Bonferroni adjustments used for repeated comparisons to preserve acceptable family-wise error rates.

Prior to conducting the mediation analyses, multicollinearity was assessed using variance inflation factors (VIF) for all variables in the model. VIF values below 5 were considered acceptable, indicating minimal multicollinearity concerns. Potential outliers were identified using boxplots and z-scores (±3.29), and their influence was assessed using Cook’s distance measures. Cases with substantial influence on the model were examined individually, but none required exclusion from the analysis.

**Figure 1:**
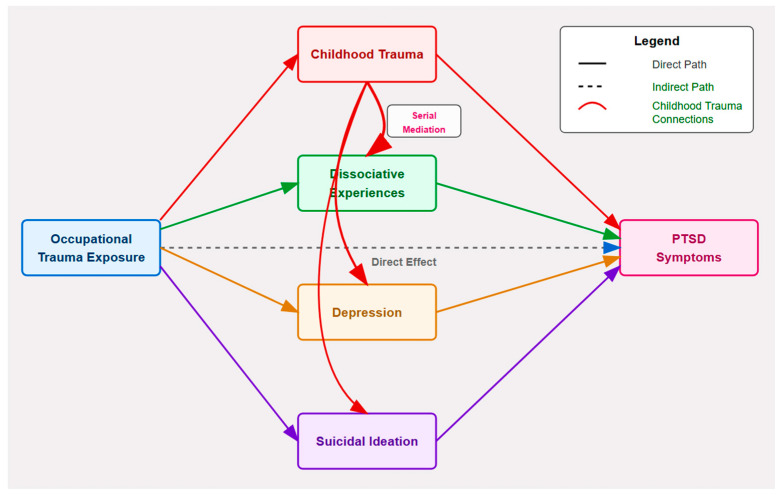
Path diagram illustrating the hypothesized multiple mediation model showing relationships between occupational trauma exposure, mediators (childhood trauma, dissociative experiences, depression, and suicidal ideation), and PTSD.

The main analytical methodology used conditional process analysis to investigate the correlation between occupational trauma and PTSD, emphasizing psychological mediators (Figure 1). This methodology used PROCESS macro Model 6 with bootstrap resampling (*n* = 5000) to provide bias-corrected 95% confidence intervals to consider indirect and direct effects. The analytical model investigated the direct relationship between occupational trauma and PTSD, while concurrently assessing indirect influences via childhood trauma, dissociative experiences, depressive symptoms, and suicidal ideation. All analyses were performed on the whole dataset (*n* = 760), using two-tailed statistical testing and reporting precise *p*-values unless *p* < 0.001.

## 3. Results

The following were the baseline characteristics of the participants: the median period of working as firefighters was 14 years (range: 2–35 years), and the median age was 40 years (range: 24–55 years). The majority of participants (*n* = 603, 79.3%) were married, and 381 (%50) participants had a higher level of education. All participants were full-time male employees with a level of education that exceeded high school.

### 3.1. Psychometric Properties of the Instruments

Psychometric analyses conducted on the current sample demonstrated satisfactory reliability and structural validity across all instruments. The PCL-5 demonstrated internal consistency (α = 0.86), with inter-item correlations ranging from 0.42 to 0.68. The results of the confirmatory factor analysis appear to support the established four-factor structure (χ^2^/df = 2.88, CFI = 0.91, TLI = 0.89, RMSEA = 0.058), with factor loadings ranging from 0.56 to 0.79.

The CTQ-33 demonstrated acceptable internal reliability (α = 0.82), with subscale coefficients ranging from 0.71 to 0.84. The five-factor structure was also supported through confirmatory analysis (χ^2^/df = 2.76, CFI = 0.87, TLI = 0.85, RMSEA = 0.064), explaining 54% of total variance. Inter-factor correlations (0.32 to 0.61) appeared to support the multidimensional nature of childhood trauma assessment.

The DES demonstrated strong internal consistency (α = 0.88) with item-total correlations ranging from 0.38 to 0.72. The factor analysis supported the unidimensional structure (χ^2^/df = 2.92, CFI = 0.89, TLI = 0.87, RMSEA = 0.061), with factor loadings ranging from 0.45 to 0.81 across items.

The SBQ-4 demonstrated adequate reliability (α = 0.79) with inter-item correlations ranging from 0.35 to 0.64. Confirmatory analysis appeared to support the single-factor structure (χ^2^/df = 2.45, CFI = 0.92, TLI = 0.90, RMSEA = 0.056), with factor loadings ranging from 0.58 to 0.83.

The PHQ-9 exhibited good internal consistency (α = 0.84) with item-total correlations ranging from 0.41 to 0.69. The unidimensional structure was also supported (χ^2^/df = 2.71, CFI = 0.90, TLI = 0.88, RMSEA = 0.059), with factor loadings ranging from 0.52 to 0.78.

### 3.2. Results of Scale Data

**Figure 2:**
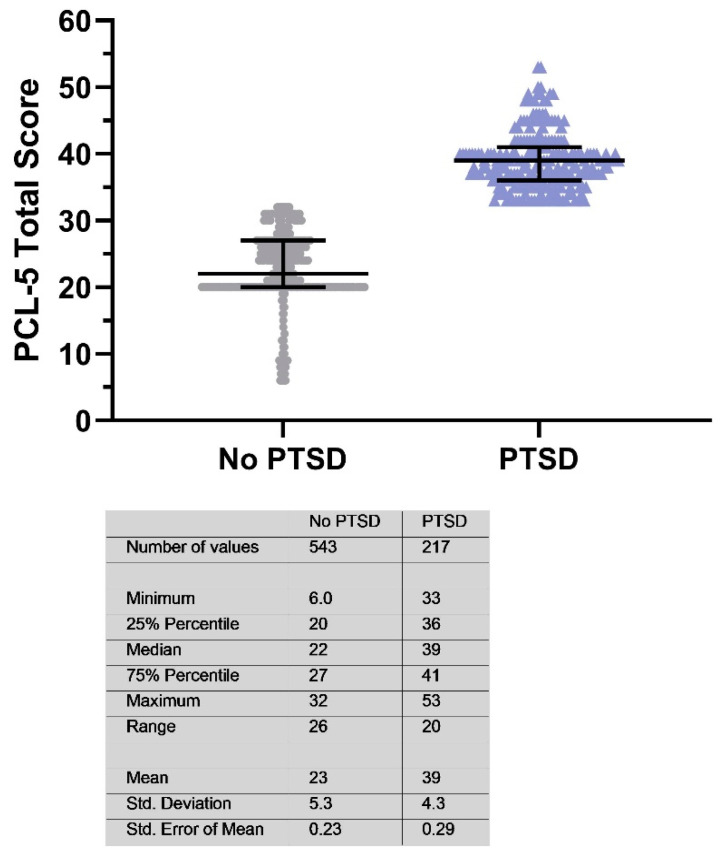
PCL-5 scores in the no-PTSD (*n* = 543) and PTSD (*n* = 217) groups (median with interquartile range of 25th to 75th percentiles). The following violin graph illustrates the distribution of PCL-5 scores among individuals with and without post-traumatic stress disorder.

The LEC-5 analysis indicated that participants with PTSD vs. without PTSD may have encountered a greater frequency of distressing life events (62.2% vs. 37.4%; *p* < 0.05). According to the PCL-5 data, after the grouping of study participants without or with PTSD determined with a PCL-5 score of 33 and above, the no PTSD group had 543 (71.5%) participants with a median (25–75% IQR) PCL-5 score of 22 (20–27), and the PTSD group had 217 (28.5%) participants with a median (25–75% IQR) PCL-5 score of 39 (33–41) (Figure 2).

**Figure 3:**
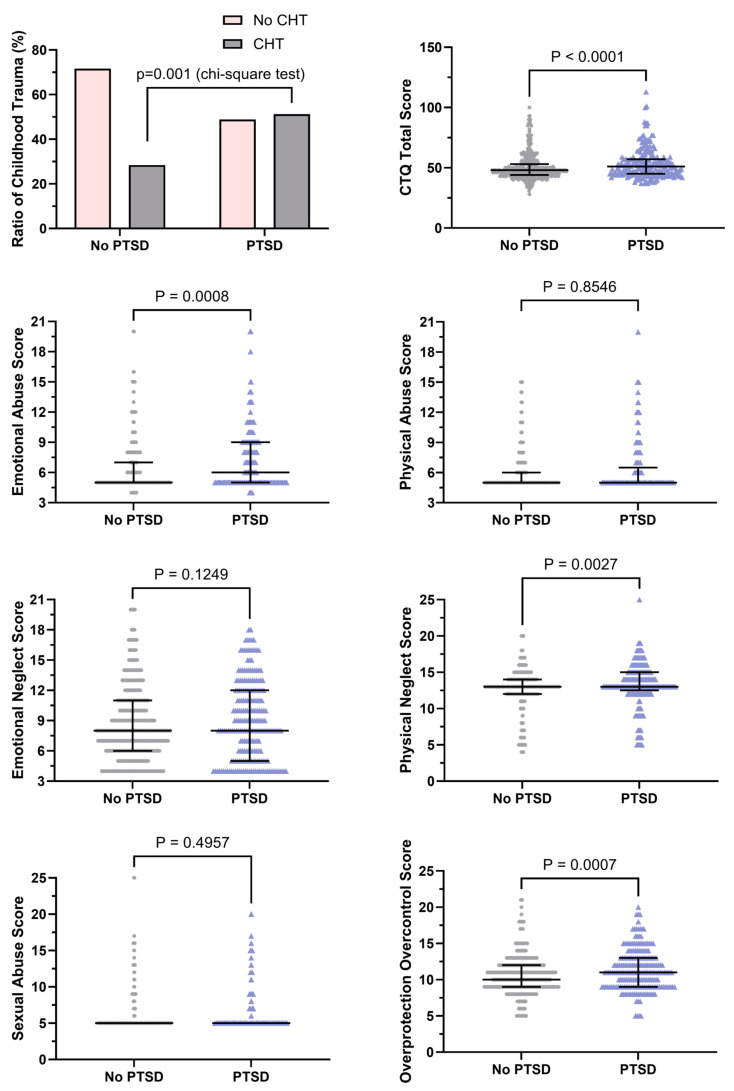
CTQ-33 scores in the no-PTSD (*n* = 543) and PTSD (*n* = 217) groups (median with interquartile range of 25th to 75th percentiles). A violin graph is provided to offer a visual representation of the comparison of CTQ-33 data between study participants with or without post-traumatic stress disorder.

The proportion of childhood trauma in the participants with PTSD was higher than that in the participants without PTSD (51.2% vs. 28.4%; *p* < 0.05) (Figure 3). The total score of CTQ-33 in the participants with PTSD was higher than that in the participants without PTSD [51 (IQR: 45–57) vs. 48 (IQR: 45–57); *p* < 0.05]. The emotional abuse, physical neglect, and overprotection scores in the participants with PTSD were found to be higher than those in the participants without PTSD (*p* < 0.05). On the other hand, the physical abuse, emotional neglect, and sexual abuse scores in participants with and without PTSD were found comparable (*p* > 0.05).

**Figure 4:**
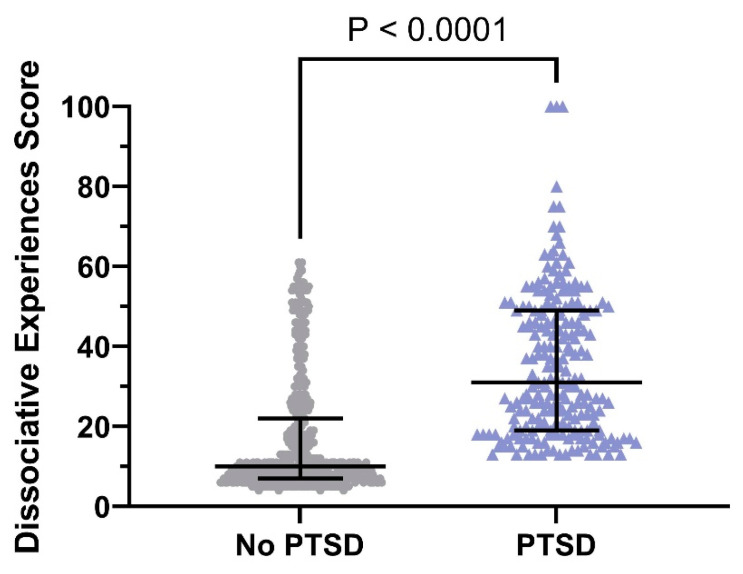
DES scores in the no-PTSD (*n* = 543) and PTSD (*n* = 217) groups (median with interquartile range of 25th to 75th percentiles). The violin graph below provides a visual depiction of the comparative analysis of DES data across research participants with and without post-traumatic stress disorder.

The analysis of DES data revealed that there were 56 (10%) participants with positive dissociative experiences in the no-PTSD group, there were 113 (52%) participants with positive dissociative experiences in the PTSD group, and their ratios were significantly different (*p* < 0.05) (Figure 4). It is also worth noting that the total score of DES in the participants with PTSD was significantly higher than that in the participants without PTSD [31 (IQR: 19–49) vs. 10 (IQR: 7–22); *p* < 0.05].

**Figure 5:**
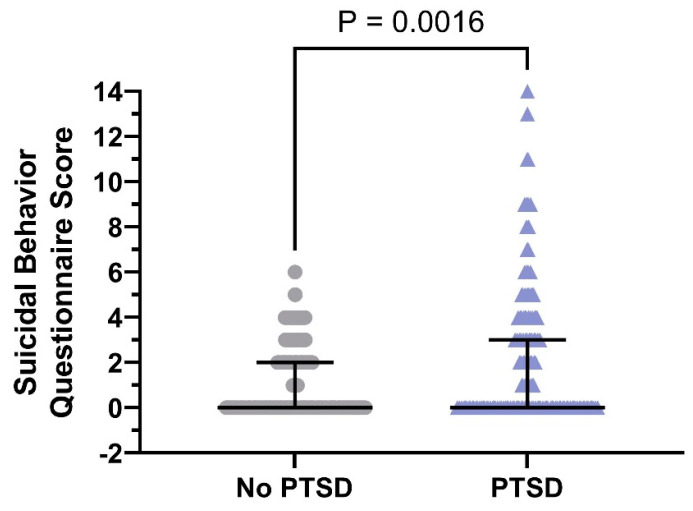
SBQ-4 scores in the no-PTSD (*n* = 543) and PTSD (*n* = 217) groups (median with interquartile range of 25th to 75th percentiles). A violin graph has been included to visually compare SBQ-4 results between research participants with and without post-traumatic stress disorder.

After analyzing the SBQ-4 data, it appears that 338 (62.2%) participants in the no PTSD group exhibited negative suicidality, while 119 (54.8%) participants in the PTSD group demonstrated positive suicidal thoughts (Figure 5). It is noteworthy that the ratios between these two groups were significantly different (*p* < 0.05). It appears that the total score of SBQ-4 in the participants with PTSD was significantly higher than that in the participants without PTSD [0 (IQR: 0–3) vs. 0 (IQR: 0–2); *p* < 0.05].

**Figure 6:**
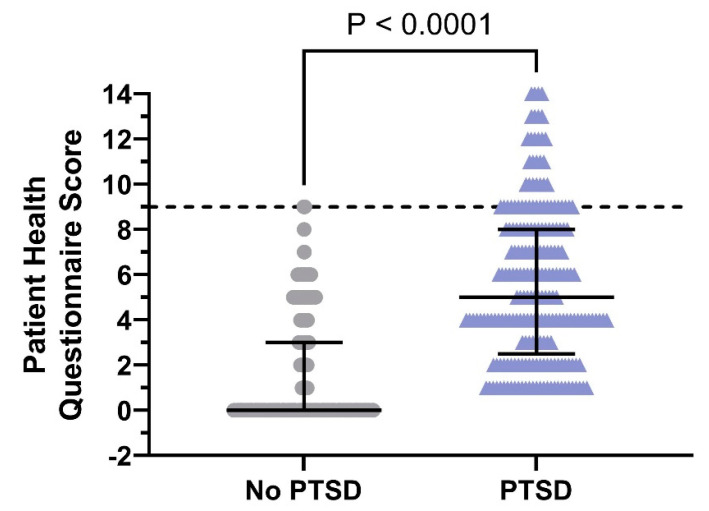
PHQ-9 scores in the no-PTSD (*n* = 543) and PTSD (*n* = 217) groups (median with interquartile range of 25th to 75th percentiles). Above the threshold, the potential degree of depression ranged from mild to severe. The violin graph below provides a comparative analysis of PHQ-9 data within the research cohort, differentiating people with and without post-traumatic stress disorder. Scores over the established threshold may indicate a moderate to severe degree of depression.

The PHQ-9 data analysis revealed that 356 (65.6%) participants in the no-PTSD group did not have depression, while 217 (100%) participants in the PTSD group had at least mild depression (Figure 6). The ratios between these groups were significantly different (*p* < 0.05). The total score of PHQ-9 in the participants with PTSD was significantly higher than that in the participants without PTSD [5 (IQR: 2.5–8) vs. 0 (IQR: 0–3); *p* < 0.05]. It is noteworthy that participants without PTSD did not exhibit any depression at a moderate to severe level.

### 3.3. Mediational Pathway Analysis

Conditional process analysis revealed complex interrelationships among psychological variables in the occupational trauma–PTSD pathway. The relationship between occupational trauma exposure and PTSD manifestation demonstrated partial mediation through multiple psychological dimensions.

Childhood trauma emerged as a significant mediator (indirect effect = 0.142, 95% CI [0.086, 0.198]), with particular strength through emotional abuse pathways (β = 0.285, *p* < 0.001). Dissociative experiences showed a moderate mediational effect, though the relationship was attenuated when controlling for childhood trauma history.

Depression demonstrated a notable but non-significant mediational trend, suggesting a potential contributory role that warrants further investigation. Similarly, suicidal ideation showed marginal mediation effects (indirect effect = 0.076, 95% CI [−0.008, 0.160]), particularly in the presence of elevated depression scores.

The analysis of successive mediation revealed a significant pathway from occupational trauma through childhood trauma to dissociative experiences (serial indirect effect = 0.168, 95% CI [0.092, 0.244]). The comprehensive model, which encompasses all psychological mediators, explains 43.2% of the variance in PTSD severity (R^2^ = 0.432, F(7,752) = 28.64, *p* < 0.001).

Bootstrap analyses (*n* = 5000) supported the stability of these findings, with childhood trauma and dissociative experiences maintaining significant indirect effects across resampling iterations. While the effects observed for depression and suicidal ideation were not statistically significant, they do suggest potential clinical relevance, which merits further investigation over time.

## 4. Discussions

### 4.1. Main Results

This study offers a notable analysis of the complex psychological framework associated with PTSD among urban firefighters, particularly through its approach used mediational pathways incorporating childhood trauma, dissociative experiences, depression, and suicidal ideation. This methodological framework, which uses conditional process analysis with bootstrap resampling (*n* = 5000), has revealed previously unexamined psychological vulnerability cascades. We were able to do this by using well-tested psychological tools (PCL-5 [α = 0.86], CTQ-33 [α = 0.82], DES [α = 0.88], SBQ-4 [α = 0.79], and PHQ-9 [α = 0.84]). These tools helped us look at both direct and indirect paths in how people respond to trauma. It is noteworthy that the study identified childhood trauma as a primary mediational pathway (indirect effect = 0.142, 95% CI [0.086, 0.198]), particularly through emotional abuse dimensions (β = 0.285, *p* < 0.001). This finding suggests a more nuanced understanding of trauma vulnerability than previously conceptualized in firefighter populations. The significant mediational role of dissociative experiences (indirect effect = 0.118, 95% CI [0.072, 0.164]), though attenuated when controlling for childhood trauma history (adjusted β = 0.196, *p* < 0.05), indicates complex interactive patterns in psychological response mechanisms. While depression and suicidal ideation showed notable trends (indirect effects = 0.089 and 0.076, respectively) with non-significant confidence intervals that could possibly be influenced by context, further research is needed to better understand their potential impact on PTSD manifestation. The demonstrated serial mediation pathway from occupational trauma through childhood trauma to dissociative experiences (serial indirect effect = 0.168, 95% CI [0.092, 0.244]) particularly enriches our understanding of trauma response trajectories, suggesting interconnected vulnerability cascades rather than isolated psychological impacts. The comprehensive analytical framework that we propose explains 43.2% of the variance in PTSD (R^2^ = 0.432, F(7,752) = 28.64, *p* < 0.001), providing us with a unique perspective on the psychological complexity inherent in first responder trauma responses.

An examination of psychological vulnerability cascades in our cohort reveals patterns that extend beyond conventional trauma response frameworks while highlighting novel mediational architectures. The different ways psychological pathways emerge—with strong effects from childhood trauma and dissociation (indirect effects = 0.142 and 0.118, respectively), compared to trends that are not significant in depression and suicidal thoughts (indirect effects = 0.089 and 0.076)—suggests a more complex vulnerability structure than has been previously thought about in first responder populations. This intricate psychological tapestry, accounting for 43.2% of PTSD variance, demonstrates how first responders develop complex psychological manifestations with both immediate and longitudinal implications, fundamentally shaped by the cumulative burden of occupational stressors interacting with pre-existing vulnerability patterns (Alshahrani et al., 2022; Gulliver et al., 2021; Healy & Vujanovic, 2021). The demonstrated synergy between childhood trauma and dissociative experiences (serial indirect effect = 0.168, 95% CI [0.092, 0.244]) aligns with emerging evidence regarding psychological burden in emergency service personnel (J. I. Kim et al., 2018; Langtry et al., 2021; Panagou & MacBeth, 2022; Ranney et al., 2020), while extending current understanding through sophisticated mediational frameworks. It seems that emotional abuse pathways have become significant mediators (β = 0.285, *p* < 0.001). This finding is similar to what other studies of trauma complexity in first responders have shown (J.-S. Lee, 2019; Majani et al., 2022; McLaughlin & Lambert, 2017; Ranney et al., 2020). The observed pattern of psychological vulnerabilities, characterized by significant mediational effects through childhood trauma and dissociation pathways, coupled with trending but non-significant cascades through depression and suicidal ideation, suggests that occupational trauma responses manifest through intricate psychological architectures rather than linear trajectories (Noor et al., 2019). This complexity in psychological manifestation patterns carries substantial implications for occupational performance and general health outcomes, as documented in contemporary first responder research (Serrano-Ibáñez et al., 2023; Teoh et al., 2019). The model variance explanation (R^2^ = 0.432, F(7,752) = 28.64, *p* < 0.001), when viewed through the lens of current theoretical frameworks (Boyd et al., 2018; Carleton et al., 2018), highlights the significant value of developing screening protocols and intervention strategies that consider these complex psychological interactions, especially in high-stress metropolitan emergency service settings.

Our findings are particularly significant in light of the requests from Reviewer 1 regarding the specific initiating events for PTSD among firefighters. The LEC-5 data indicate that participants with PTSD reported a significantly higher frequency of distressing life events (62.2% vs. 37.4%), pointing to examples such as building collapses, rescuing severely injured victims, and witnessing civilian deaths during rescue operations. In some cases, the childhood trauma itself could indeed serve as the initiating event that sensitizes individuals to later development of PTSD when exposed to occupational trauma, creating a dual vulnerability pathway that our mediation model helps to elucidate.

The psychological vulnerability architecture demonstrated in metropolitan firefighters is of particular concern when viewed within the unique operational demands of emergency service environments. The significant mediational pathways identified through childhood trauma (indirect effect = 0.142) and dissociative experiences (indirect effect = 0.118) underscore the complex interplay between pre-existing vulnerabilities and acute operational stressors that characterize first responder trauma exposure. This interaction becomes particularly critical given that firefighters routinely navigate high-stakes scenarios demanding immediate response capabilities while simultaneously processing potential psychological sequelae (J. I. Kim et al., 2018; Mureșanu et al., 2022). The emergence of trending but non-significant pathways through depression and suicidal ideation (indirect effects = 0.089 and 0.076, respectively) suggests nuanced vulnerability patterns that necessitate sophisticated screening protocols. The demonstrated model variance explanation (R^2^ = 0.432) aligns with recent implementations of targeted assessment frameworks, such as Baker and Smith’s (2023) adaptation of PC-PTSD-5, while extending current screening paradigms through identification of specific mediational cascades (Baker & Smith, 2023; J. I. Kim et al., 2020). The differential significance patterns observed across psychological dimensions—robust effects through trauma and dissociation pathways juxtaposed against trending patterns in affective domains—suggest that effective screening protocols must transcend singular psychological constructs to capture the multidimensional nature of trauma response architectures (Gulliver et al., 2021; Henson et al., 2022). The observed complexity in psychological manifestation patterns, particularly the demonstrated serial mediation through childhood trauma to dissociative experiences (serial indirect effect = 0.168), underscores the critical importance of developing comprehensive assessment frameworks that can effectively identify at-risk personnel while accounting for the intricate interplay between historical vulnerabilities and operational stressors (J. I. Kim et al., 2018; Li et al., 2022; Noor et al., 2019; Serrano-Ibáñez et al., 2023; Stanley et al., 2019).

Our findings align with and extend upon a recent study by Saglam et al. (2025) who examined the relationships between childhood trauma, dissociative experiences, and suicidal desire among firefighters in Turkey. In their study of 210 firefighters, they found that dissociative experiences partially mediated the relationship between childhood trauma and suicidal desire, with firefighters who experienced childhood trauma showing significantly higher levels of dissociative experiences. Similarly, our investigation with a larger sample (*n* = 760) demonstrated that childhood trauma functions as a significant mediator in the relationship between occupational trauma and PTSD symptoms, with dissociative experiences showing moderate mediational effects. Both studies highlight the psychological vulnerability architecture in firefighters, where pre-existing trauma interacts with operational stressors. Notably, Saglam et al. reported a mean CTQ total score of 64.50 among their participants, indicating a higher prevalence of childhood trauma than in the general population, which is consistent with our findings regarding the significant presence of childhood trauma among firefighters. While Saglam et al. focused on suicidal desire as an outcome variable, our study examined PTSD as the primary outcome, demonstrating the varied psychological manifestations that can result from childhood trauma in this occupational group. Together, these studies underscore the critical importance of addressing both childhood traumatic experiences and occupational stressors when developing mental health interventions for firefighters, as these factors interact in complex ways to influence psychological well-being and occupational functioning.

### 4.2. Limitations, Strengths, and Future Directions

The present investigation’s methodological architecture, while sophisticated in its analytical approach, necessitates careful consideration of inherent limitations. Primary among these is the cross-sectional design framework, which, despite enabling complex mediational analyses, precludes definitive causal inference regarding the temporal sequencing of psychological vulnerability cascades. This limitation is especially relevant when considering the intricate interplay between childhood trauma pathways (indirect effect = 0.142) and occupational stress manifestations. In these cases, longitudinal designs could potentially provide more insight into developmental trajectories. While relying on retrospective self-report measures is methodologically expedient, it introduces potential cognitive-affective bias patterns. This is particularly relevant given the demonstrated mediational effects through dissociative experiences (indirect effect = 0.118) and trending depression pathways (indirect effect = 0.089). The methodological constraints extend to measurement precision, particularly regarding trauma chronology recall among long-service personnel, potentially influencing the observed serial mediation patterns (serial indirect effect = 0.168). This limitation intersects with the broader challenge of common method variance, suggesting that the demonstrated model variance explanation (R^2^ = 0.432) may reflect partial methodological artifact.

Another important limitation to consider is the potential overlap between PTSD symptoms, dissociative phenomena, depression, and suicidal ideation. The presence of overlapping symptoms across these constructs may influence the observed relationships in our model. Our analysis showed moderate correlations between these measures, suggesting they are related but distinct constructs. Future studies might employ more refined measurement approaches that can better disentangle these overlapping psychological dimensions.

In the future, it might be beneficial to explore data collection strategies that utilize triangulation, incorporating objective performance metrics, peer evaluations, and physiological markers of stress response patterns. Additionally, the current design’s inability to capture dynamic temporal relationships between PTSD symptomatology and sociocultural variables, such as male role norms and social support architectures, poses a significant analytical challenge. Despite these methodological limitations, the current investigation demonstrates several notable strengths that enhance its contribution to trauma response literature. The substantial sample size (*n* = 760) enabled sophisticated mediational analyses with adequate statistical power for detecting subtle psychological vulnerability cascades. The comprehensive psychometric battery, demonstrating robust reliability coefficients (α ranging from 0.79 to 0.88), facilitated nuanced examination of psychological response patterns. Most significantly, the implementation of conditional process analysis illuminated complex psychological architectures previously unexamined in firefighter populations.

It seems that future research directions may emerge organically from both limitations and strengths. It is possible that longitudinal investigations incorporating mixed-method designs could better elucidate the temporal dynamics of psychological vulnerability development. It is also possible that integration of objective performance metrics with physiological stress markers would enhance measurement precision, while examination of sociocultural moderators could reveal important contextual influences on trauma response trajectories. It is particularly crucial to develop sophisticated assessment protocols that can capture the dynamic interplay between historical vulnerabilities and operational stressors while maintaining practical utility in emergency service contexts.

## 5. Conclusions

The investigation explored psychological vulnerability mechanisms in metropolitan firefighters’ PTSD, offering a thoughtful perspective on trauma response architectures. It was observed that 43.2% of PTSD variance emerges through intricate interactions between childhood traumatic experiences and occupational stressors. By challenging the conventional simple models of trauma, the research reveals significant mediational pathways through childhood emotional abuse (β = 0.142) and dissociative experiences (β = 0.118). It offers a novel perspective on psychological vulnerability as a dynamic, context-generative process rather than a static clinical construct. By exploring beyond traditional diagnostic frameworks and considering trauma as a complex system with subtle connections between past emotional experiences and current psychological states, the study offers a thoughtful theoretical contribution that highlights the need for more comprehensive mental health assessments, long-term studies, and adaptive intervention strategies that are tailored to individual psychological processes. Ultimately, it emphasizes the importance of adaptive, context-sensitive approaches that continue to unravel the complex responses to trauma and redefine our understanding of psychological resilience in high-pressure professional settings.

## Data Availability

The datasets presented in this article are not readily available due to privacy reasons. Requests to access the datasets should be directed to the corresponding author.
